# Does structural leg-length discrepancy affect postural control? Preliminary study

**DOI:** 10.1186/s12891-017-1707-x

**Published:** 2017-08-09

**Authors:** Małgorzata Eliks, Wioleta Ostiak-Tomaszewska, Przemysław Lisiński, Paweł Koczewski

**Affiliations:** 1 Department of Health Prophylaxis, Laboratory of Medical Electrodiagnostics, Poznan University of Medical Sciences, Heliodor Swiecicki Clinical Hospital, 49 Przybyszewskiego St., 60-101 Poznań, Poland; 2 Department of Paediatric Orthopaedics and Traumatology, Poznan University of Medical Sciences, Wiktor Dega Orthopaedic and Rehabilitation Clinical Hospital, 135/147 28 Czerwca 1956 r. St., 61-545 Poznań, Poland; 3 Department of Rehabilitation, Poznan University of Medical Sciences, Wiktor Dega Orthopaedic and Rehabilitation Clinical Hospital, 135/147 28 Czerwca 1956 r. St., 61-545 Poznań, Poland

**Keywords:** Leg-length inequality, Postural balance, Weight bearing, Postural control, Weight distribution

## Abstract

**Background:**

Leg-length inequality results in an altered position of the spine and pelvis. Previous studies on the influence of leg asymmetry on postural control have been inconclusive. The purpose of this paper was to investigate the effect of structural leg-length discrepancy (LLD) on the control of posture.

**Methods:**

We studied 38 individuals (19 patients with structural LLD, 19 healthy subjects). The examination included measurement of the length of the lower limbs and weight distribution as well as a static posturography. All statistical analyses were performed with Statistica software version 10.0. Non-parametrical Kruskal-Wallis with Dunn’s post test and Spearman test were used. Differences between the groups and correlation between mean COP sway velocity and the value of LLD as well as the value of LLD and weight distribution were assumed as statistically significant at *p* < 0.05.

**Results:**

There was a significant difference in the asymmetry of weight distribution between the group of patients and the healthy subjects (*p* = 0.0005). Differences in a posturographic examination between the groups were not statistically significant (*p* > 0.05). Meaningful differences in mean COP velocity in mediolateral direction between tandem stance with eyes open and closed were detected in both groups (in controls *p* = 0.000134, in patients both with the shorter leg in a front and rear position, *p* = 0.029, *p* = 0.026 respectively). There was a positive moderate correlation between the value of LLD and the value of mean COP velocity in normal standing in mediolateral direction with eyes open (*r* = 0.47) and closed (*r* = 0.54) and in anterioposterior plane with eyes closed (*r* = 0.05).

**Conclusions:**

The fact that there were no significant differences in posturography between the groups might indicate compensations to the altered posture and neuromuscular adaptations in patients with structural leg-length inequality. LLD causes an increased asymmetry of weight distribution. This study confirmed a fundamental role of the sight in postural control, especially in unstable conditions. The analysis of mean COP sway velocity may suggest a proportional deterioration of postural control with the increase of the value of leg-length asymmetry.

**Trial registration number:**

Trial registry: ClinicalTrials.gov NCT03048656, 8 February 2017 (retrospectively registered).

## Background

Structural leg-length inequality caused by the shortening of a segment of the extremity, results in an altered position of the lower limb joints, the pelvis and the spine in static as well as dynamic conditions [1–5]. That may induce a disturbance of postural control, which could increase the risk of potential falls in patients undergoing a lengthening procedure with external fixation methods. Detecting these deficits would indicate the need of including balance exercises to a physical therapy management in order to prevent injuries and complications.

Structural leg-length discrepancy (LLD) is the result of congenital and developmental disorders or a damage of the epiphyseal plate in the consequence of an injury or joint inflammation [6]. The of study by Drnach et al. showed that LLD greater than 2 cm occurs in 7% of healthy children (8–12 years of age) [7]. It should be emphasised that even as small asymmetry as 2 cm seems to be clinically meaningful [6, 8]. It was noticed that this value is a threshold for compensation of the pelvis to postural imbalance [8]. What is more, the discrepancy of 2 cm influences the gait economy by the increase of oxygen consumption and the rating of perceived exertion as well [6].

In recent years, there has been an increasing interest in determining the influence of LLD on the position of the spine and pelvis. Betsch et al. [2] observed that simulated LLD smaller than 15 mm caused pelvic tilt and pelvic torsion, however, no significant changes in the position of the spine were detected. The studies by Betsch et al. [1], Young et al. [5] revealed that artificial LLD greater than 15 mm caused lateral flexion of the trunk (evoking functional scoliosis), the asymmetry of more than 20 mm resulted in a significant increase of the obliquity and the torsion of the pelvis as well as a clear increase of the surface rotation and lateral deviation of the spine. The consequence of induced LLD greater than 50 mm was a significant increase in pelvic inclination [1].

Lower limb length asymmetry elicits an alternation of muscle activity. Abate et al. [9] performed an investigation with a high resolution thermal infrared imaging in subjects with simulated LLD. The findings showed a greater activity in the muscles of both extremities: tibialis anterior, gastrocnemius, quadriceps femoris and latissimus dorsi [9]. Furthermore, the experimental LLD amplified the activity of lower extremity muscles and intrinsic lumbar back muscles in an electromyography during gait [3, 10]. To the best of our knowledge there has been no study on muscle activity with the use of electromyography in standing in patients with structural LLD. Taking into consideration the above-mentioned sentences, it seems obvious that posture of the individuals with LLD is disturbed.

Postural control is a complex motor skill whose goal is to provide stability in a gravity field by maintaining the centre of mass over the base of support [11, 12]. The nervous and the musculoskeletal systems cooperate to provide postural orientation and equilibrium, for instance by anticipation of any destabilisations or reaction to perturbations [11, 12]. Anticipatory adjustments are based on a feedforward mechanism, whereas postural reactions are controlled by a feedback regulation and include movement strategies which are dependent on a postural alignment [11, 13, 14].

Visual, vestibular, somatosensory inputs received by receptors of sensory systems are fundamental in postural control [11, 14, 15]. It was observed that visual deprivation resulted in a greater sway of the centre of pressure (COP) [16]. This phenomenon might be explained by the absence of anticipatory postural adjustments (APAs)’ generation without visual inputs [17]. The ability to control COP position determines correct stability of posture.

The sway of COP in standing can be registered and analysed by a static posturography [14, 18, 19]. The posturographic examination measures biomechanical parameters such as: symmetry of weight distribution, COP sway amplitude, COP sway velocity, COP path sway [18, 20]. The measurement of parameter of the COP sway velocity is clinically useful, because it was found that an increase in COP sway velocity indicates disturbances of postural control [20, 21].

The purpose of this study was to investigate the effect of structural LLD on the control of posture. We hypothesised that LLD causes an asymmetry of weight distribution and a deterioration of postural control, especially in condition of a visual deprivation and a narrowed width of the base of support. We assumed also that the value of LLD correlates with the deterioration of postural control. Previously, few studies [4, 22] on the influence of leg length asymmetry on postural control have been inconclusive, therefore investigating this topic of the research seems to be justified.

## Methods

The study included 38 participants (19 patients in the experimental group, 19 volunteers in the control group). All subjects or their caregivers expressed a written consent to the examination.

The experimental group included 8 women and 11 men (at the mean age of 14 years, SD = 6.59, min = 6 years, max = 30 years) – patients of Department of Paediatric Orthopaedics and Traumatology, Poznan University of Medical Sciences. All patients were qualified to a lengthening procedure with external fixation methods (the Ilizarov apparatus or a monolateral fixator). The shortening of left lower limb occurred in 12 patients, of the right extremity in 7 patients. Mean value of leg length asymmetry was 5.8 cm (SD = 3.08, min = 2 cm, max = 13 cm). Congenital etiology of LLD caused by for instance fibular hypoplasia and innate femur shortening occurred in 12 individuals, 7 patients acquired LLD as a result of such conditions as injury, Perthes’ disease, knee or hip arthritis in their childhood. Exclusion criteria from experimental group involved: achondroplasia, non-union, idiopathic scoliosis, extremity shortening in the course of neurological disease (e.g. Cerebral Palsy), vestibular disorder (e.g. Ménière’s disease), diabetes, sensory disorder, intake of medications affecting psychomotor activity, dizziness, neurological diseases (e.g. epilepsy), BMI > 30, using mobility aids (e.g. crutches).

The control group included 11 women and 8 men (mean age of 18 years, SD = 5.49, min = 6 years, max = 26 years). Exclusion criteria from the control group were: leg-length discrepancy, scoliosis, faulty posture, vestibular disorders, diabetes, intake of medications affecting psychomotor activity, dizziness, sensory disorders, neurological disease, BMI > 30.

The study was conducted in the agreement with the Declaration of Helsinki and with the approval of the Bioethics Committee of Poznan University of Medical Sciences (the reference number 418/14).

The examination of participants included the measurement of the length of the lower limbs and a weight distribution as well as performing a static posturography.

The leg length was measured with the use of indirect method [23]. In standing, wooden blocks were placed under the shorter leg in order to even the position of both anterior superior iliac spines [23]. The height of the blocks determined the value of a leg length asymmetry [23].

The measurement of a weight distribution and a static posturography was performed on a balance platform Good Balance by Metitur (Jyväskylä, Finnland). The device comprises of a triangular force platform (800 mm × 800 mm × 800 mm) with an electronic system and a computer software. The sampling frequency was 50 Hz. The measurement error in the calculated mediolateral and anteroposterior coordinates of the COP was +/− 1.0 mm or +/−1.2 mm, if the mass of the individual was at least 40 kg or 30 kg respectively. Body weight distribution was evaluated in an upright standing with eyes open, feet placed 20 cm from each other [21] or narrower in children, with the upper extremities in a relaxed position by the sides. The individual stood motionlessly for 15 s for stabilising the measurement result displayed on the monitor, which was visible only for the investigator. Then the test stopped and the outcome was recorded. The results of each lower extremity loading and a difference in weight bearing between extremities were expressed in percentages (%) [21].

A static posturography examination was performed with 3 various positions of feet, both with eyes open (EO) and eyes closed (EC). (1) Position [24]: *normal standing* – an upright standing with feet placed parallel 20 cm apart or narrower in children. (2) Position: *tandem* [24] – a stance with 1 foot placed ahead of the other, medial edge of feet was put on the midline of the balance platform. In the experimental group 2 trials were recorded: (a) foot of the shorter leg in the front, (b) foot of the shorter leg in the rear. In the control group also 2 trials were performed: (c) foot of the right leg in the front, (d) foot of the left leg in the front. (3) Position*: one leg standing*: stance on one leg, foot placed 10 cm from midline of the platform, the other – 90 degrees flexion of the knee and the hip, test performed for each leg, only with EO. Every participant was supposed to stand motionlessly for 30 s in *normal standing* [21, 24, 25] or for 20 s in *tandem* [21, 24, 25] and *one leg standing* position. Conditions of the posturographic examination involved: a quiet and normally lit room [4], standing barefoot [26], the eyesight directed at a point in the distance of 2 m [25], glasses or contact lenses were worn if they were normally needed, arms held in the front of the body with hands together in order to limit movements of the upper extremities [25]. The recording was initiated when a stable position was attained [25]. Each test was performed once [25].

According to literature [27] a weight distribution measurement was treated in this study as a preliminary evaluation, that could indicate a postural control deficiency. Mean velocity (mm/s) of COP sway was measured as a quantitative parameter of a postural control assessment [28]. Mean COP sway velocity was recorded both in anteroposterior (AP) and mediolateral (ML) directions [24–26].

Data were given as mean value, minimum, maximum and standard deviation. All statistical analyses were performed with Statistica software version 10.0. Non-parametrical Kruskal-Wallis with Dunn’s post-test and Spearman test were used. The differences between the groups and correlation between the mean COP sway velocity and the value of LLD as well as the value of LLD and weight distribution were assumed as statistically significant at *p* < 0.05.

## Results

No significant differences (*p* > 0.05) were found between loading of the shorter and the longer leg in the experimental group and between loading of the right and the left extremity in the control group (Table 1). Nevertheless the differences in weight bearing of limbs between the groups were significant (*p* < 0.05) (Table 1).

**Table 1:** The results of measurements of weight distribution of each lower limb and their differences in the experimental group and the control group. The results of each lower extremity loading and a difference in weight bearing between extremities were expressed in percentages [%]

Variable	Weight distribution				Differences in weigth distribution	
	Experimental group		Control group		Experimental group	Control group
	Shorter limb	Longer limb	Right limb	Left limb		
Mean [%]	51.63	48.37	51.74	48.26	17.33^#^	5.16
Min [%]	32.00	22.00	46.00	42.00	2.00	0.0
Max [%]	78.00	68.00	58.00	54.00	56.00	16.00
SD	11.25	11.25	3.26	3.26	14.16	4.26

^#^ *p* < 0.05

The measurement of weight distribution in the experimental group showed that 10 individuals shifted body weight to the shorter leg (>50%), 9 patients to the longer limb (≥50%).

Statistical analyses with the use of Spearman’s rank correlation coefficient did not reveal a correlation between the value of leg length inequality and differences in a load distribution between the shorter and the longer limb in the experimental group (*r* = 0.09, *p* = 0.71).

The mean COP sway velocity in *normal standing, tandem* and *one leg standing* in AP and ML directions both with eyes open and closed was higher in the group of patients than in the healthy subjects, however, the differences were not significant (*p* > 0.05) (Table 2).

**Table 2:** The results of the static posturography of the experimental group and the control group in *normal standing, tandem*, *one leg standing*, with open (EO) and closed eyes (EC). No significant differences were found

Variable	Normal standing EO		Normal standing EC		Tandem, the shorter limb in the front position EO		Tandem, the shorter limb in the front position EC		Tandem, the shorter limb in the rear position EO		Tandem, the shorter limb in the rear position EC		One leg standing on the shorter limb		One leg standing on the longer limb	
	Group A^a^	Group B^b^	Group A	Group B	Group A	Group B	Group A	Group B	Group A	Group B	Group A	Group B	Group A	Group B	Group A	Group B
Mean COP sway velocity [mm/s] ML (SD)	9.5 (6.4)	4.6 (5.0)	18.5 (21.6)	5.8 (7.8)	16.9 (11.7)	13.6 (7.5)	32.3 (13.1)	29.0 (18.6)	19.1 (12.8)	13.6 (7.5)	41.9 (25.3)	29.0 (18.6)	45.4 (35.8)	23.5 (11.1)	27.5 (20.9)	23.5 (11.1)
Mean COP sway velocity [mm/s] AP (SD)	12.1 (9.1)	6.3 (5.7)	19.1 (11.8)	8.6 (7.1)	22.1 (20.1)	16.1 (12.9)	32.2 (18.7)	28.9 (34.6)	31.6 (38.5)	16.1 (12.9)	47.1 (29.0)	28.9 (34.6)	48.5 (39.5)	22.5 (18.0)	28.3 (24.7)	22.5 (18.0)

^a^Group A – individuals with LLD

^b^Group B – healthy individuals

There was no significant difference in mean COP sway velocity between the right and left foot in the front position in *tandem* stance in the control group. Thus the results of *tandem* with the right foot forward were chosen to comparison with the experimental group. Two patients were not able to perform the test in *tandem* with EC with the shorter leg in the back. No significant difference was found in the mean COP sway velocity between *one leg standing* on the right and on the left foot in the control group. Therefore, the results of *one leg standing* test on the right foot were selected to comparison with the experimental group. Five patients were not able to perform the test.

The results of the measurements of the mean COP sway velocity in *tandem* position with EO and EC are presented in Table 3.

**Table 3:** The analysis of the mean COP sway velocity in *tandem* position, in both groups, with open (EO) and closed eyes (EC) with significant differences of the mean COP sway velocity with EO and EC

Variable	Tandem, the shorter limb in the front position		Tandem, the shorter limb in the rear position		Tandem	
	Group A^a^		Group A		Group B^b^	
	EO	EC	EO	EC	EO	EC
Mean COP sway velocity ML [mm/s]	16.9	32.3^c^	19.1	41.9^#^	13.6	29.0^#^
Mean COP sway velocity AP [mm/s]	22.1	32.2	31.6	47.1	16.1	28.9

^a^Group A – individuals with LLD

^b^Group B – healthy individuals

^#^p (EC–EO) < 0.05

Significant differences were noticed in mean COP sway velocity in the ML plane between *tandem* with EO and EC in both groups. In the AP direction no significant differences were found. In the group of patients there was a significant difference in mean COP sway velocity between the tests with EO and EC in *tandem* both with the shorter leg forward and backward (*p* < 0.05). In the control group the difference in mean COP sway velocity between the trials with EO and EC was also significant (*p* < 0.05).

Statistical analyses with the use of Spearman’s rank correlation coefficient detected a moderate positive correlation between the value of LLD and mean COP sway velocity in ML direction in *normal standing* with eyes open (*p* = 0.04, *r* = 0.47) as well as between the value of LLD and mean COP sway velocity in ML (*p* = 0.02, *r* = 0.54) and AP (*p* = 0.03, *r* = 0.50) directions in *normal standing* with eyes closed (Table 4).

**Table 4:** The analysis of correlation between the value of LLD and mean COP sway velocity in *normal standing*, with open (EO) and closed eyes (EC)

Variable	Normal standing					
	Mean COP sway velocity in ML				Mean COP sway velocity in AP	
	EO		EC		EC	
	p	r	p	r	p	r
The value of LLD	0.04	0.47	0.02	0.54	0.03	0.50

No correlations were found in other positions (*p* > 0.05).

## Discussion

We hypothesised that structural LLD influences considerably posture stability.

In every trial of the posturography in the group of patients the mean COP sway velocity was higher than in the control group. It could indicate a difficulty in maintaining upright position within individuals with LLD in imposed conditions of a posturographic examination. However, no statistically significant differences in mean COP sway velocity were found between the groups. We assume that it might be the effect of an increased resting activity of the lower limb muscles in individuals with LLD during posturographic tests. But this hypothesis requires a verification and further research with the use of electromyography. No specific differences in postural control between both groups might be explained by the fact that LLD did not constrain a participation in physical education classes or recreational physical activities.

Our results are consisted with the findings of the study by Murrel et al. [22] which revealed no differences in a posturographic evaluation between a group with structural LLD (mean value of asymmetry = 11.3 mm) and a healthy group. The authors interpreted this finding by an occurrence of a neuromuscular adaptation to the changes in posture induced by leg length asymmetry.

The proportional increase in displacements of COP in *normal standing* in the ML direction with EO and in AP and ML directions with EC was detected with the increase of the value of LLD. Mahar et al. [4] also observed correlative increase in COP sway with the growth of value of simulated LLD.

What is worth noticing, significant differences in the mean COP sway velocity in the ML plane between trials with EO and EC in *tandem* position were found in both groups. This finding might indicate a limited compensation of proprioception in the condition of narrow width of the base of support and visual deprivation. That can be also an evidence that vision cues have a predominant role in maintaining static balance in challenging conditions. This observation confirmed the results of studies on fundamental role of sight in postural control, especially in an unstable position [29, 30]. The previous studies revealed greater COP sway in the ML direction than in the AP direction in *tandem*, and also an increase in COP displacement after closing the eyes [29, 30]. Sozzi et al. [30] performed the electromyography in healthy individuals in *tandem* position. The examination detected a tonic activity in both soleus muscles and an increased muscle activity of the lower limb placed in the rear position [30]. This finding might explain a difficulty in maintaining *tandem* position with the shorter leg backward. Moreover, it was proved that standing in *tandem* is an attention-demanding task [29].

This study revealed an individual preference of an increased loading of the shorter or the longer leg. This phenomenon did not correlate with the value of lower limbs asymmetry. Raczkowski et al. [31] observed that small LLD coexisting with functional scoliosis cause the shift of body weight towards the longer leg. The research by Mahar et al. [4] also showed that simulated LLD induced greater COP shift to a longer extremity. The contrary results were presented by Swaminathan et al. [22], the artificial LLD resulted in the increased weight bearing of a shorter extremity. We assumed that contrary results may arise from various value of LLD in the above mentioned studies – Raczkowski et al. (0.5-2 cm), Swaminathan et al. (3.5 cm), Maher (1-4 cm). According to the mentioned data on minor [31] or artificial [4, 32] LLD, the comparison of our results with existing literature is troublesome.

We acknowledge the limitations of our study due to its pilot character. Our investigation did not reveal any particular disturbances of postural control in patients with LLD. Hence we insist on the complementation of static posturography with electromyography in order to reveal patterns of muscle activity which are likely to serve as a compensation to posture alternations. The examined group should be more numerous and more homogenous in terms of value of LLD and age in a further stage of the research on the influence of LLD on postural control.

## Conclusions

No significant disturbances of postural control in individuals with LLD were found in the presented study. That can support the thesis about posture compensations caused by a neuromuscular adaptation. The results of the investigation show that LLD causes a significant increase in the asymmetry of weight distribution. However, the preference of a greater limb loading (the shorter or the longer leg) is an individual feature. Due to the fact that an appropriate weight bearing is crucial for a correct osteogenesis process in the course of a leg lengthening procedure with the external fixation methods, the measurement of the symmetry of weight distribution is particularly essential at every stage of the treatment. The study confirms the important role of visual inputs in a feedback control of posture, especially in the condition of a narrowed width of the base of support. This observation does not depend on an occurrence of LLD. The analysis of mean COP sway velocity may suggest a proportional deterioration of postural control with the growth of the value of LLD.

## Abbreviations

AP: Anteroposterior
BMI: Body Mass Index
COP: Centre of pressure
EC: Eyes closed
EO: Eyes open
LLD: Leg-length discrepancy
ML: Mediolateral
